# The sex‐dependent role of the androgen receptor in glioblastoma: results of molecular analyses

**DOI:** 10.1002/1878-0261.13262

**Published:** 2022-06-22

**Authors:** Małgorzata Łysiak, Małgorzata Trybuła, Munila Mudaisi, Charlotte Bratthäll, Michael Strandeus, Peter Milos, Martin Hallbeck, Annika Malmström

**Affiliations:** ^1^ Department of Biomedical and Clinical Sciences Linköping University Sweden; ^2^ Department of Pharmaceutical Biochemistry Poznan University of Medical Sciences Poland; ^3^ Department of Oncology Linköping University Hospital Sweden; ^4^ Department of Oncology District Hospital Kalmar Sweden; ^5^ Department of Oncology Ryhov Hospital Jönköping Sweden; ^6^ Department of Neurosurgery Linköping University Hospital Sweden; ^7^ Department of Clinical Pathology Linköping University Hospital Sweden; ^8^ Department of Advanced Home Care Linköping University Sweden

**Keywords:** androgen receptor, glioblastoma, sex differences

## Abstract

We sought to analyse the androgen receptor (AR) in glioblastoma (GBM) due to the location of the *AR* gene on chromosome X, often reported with shorter survival and higher prevalence of GBM among males. Copy number (CN) and mRNA expression of AR were tested with droplet digital PCR in 91 fresh‐frozen GBM samples and 170 formalin‐fixed, paraffin‐embedded samples collected at Linköping University Hospital. The fresh‐frozen cohort was also subjected to pyrosequencing methylation analysis of 17 CpG sites in the *AR* promoter. Additionally, the gene expression of AR was analysed in the fresh‐frozen cohort and The Cancer Genome Atlas (TCGA) cohort of isocitrate dehydrogenase wild‐type primary GBM (135 females and 219 males). The association of AR expression and overall survival (OS) was tested with Kaplan–Meier log rank analysis after dichotomisation by maximally selected rank statistics. We found that AR CN alterations were more common in female GBM. AR gene expression correlated with methylation levels of different CpG sites in males and females but there was no difference in expression between sexes. Survival analysis of TCGA cohort revealed the opposite effect of AR overexpression on OS of males and females, with high AR expression correlating with shorter OS in females and longer OS in males. Additional gene set enrichment analysis showed that AR expression correlated with DNA repair response, especially in the male group. In summary, we found that high *AR* gene expression in GBM exhibits sex‐dependent effects on patient survival, which, for males, is linked to DNA repair response.

## Graphical abstract

Glioblastoma (GBM) is more common among males; however, the reason for sex differences remains largely unknown. Through molecular analyses of androgen receptor (AR) in GBM samples, we found that AR is commonly expressed in tumours of both sexes, but copy number alterations of the gene coding for AR are more frequent in females. Additionally, high AR expression correlates with worse survival among females but better survival among males.

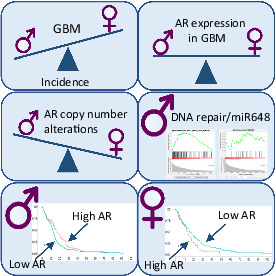

**Abbreviations**
Akt: protein kinase B
AR: androgen receptor
ATRX: alpha thalassemia/mental retardation syndrome X‐Linked
CN: copy number
DDX3X: DEAD‐box helicase 3 X‐linked
DHT: dihydrotestosterone
EGFR: epithelial growth factor receptor
FDR: false discovery rate
FFPE: formalin‐fixed, paraffin‐embedded
GBM: glioblastoma
GSEA: gene set enrichment analysis
IDH: isocitrate dehydrogenase
MGMT: *O*‐6‐methylguanine‐DNA methyltransferase
miR: microRNA
mTOR: mechanistic target of rapamycin kinase
OS: overall survival
PI3K: phosphoinositide 3‐kinase
SVZ: subventricular zone
TCGA: The Cancer Genome Atlas
TCPA: The Cancer Proteome Atlas
TGFβ: transforming growth factor β

## 1 Introduction

Glioblastoma (GBM) remains a nearly incurable brain tumour with a poor prognosis (median survival of 1.2 years) [1], despite enormous efforts and advances in knowledge. Interestingly, females have been reported to have survival advantage, and male sex is often a negative prognostic factor in GBM [2]. The effect of sex can also be observed in the incident data, with the male to female ratio being 1.6 : 1 [3]. GBM belongs to molecularly very well characterised tumours [4, 5, 6], however, the background of sex differences remains largely unknown, and clinical trials commonly neglect to investigate them.

Sex chromosomes are often purposefully omitted in analyses, due to the challenges they present. The gene coding for the androgen receptor (AR) is located on chromosome X, Xq12. The AR is a ligand‐dependent nuclear transcription factor and a member of a superfamily of steroid hormone nuclear receptors. Androgens, testosterone and dihydrotestosterone (DHT), mediate their effects via AR, and when bound to cytoplasmic AR cause the release of heat shock proteins [7]. The ligand binding also induces phosphorylation of AR and dimerisation, which finally lead to translocation and binding to DNA. The ligand binding domain has C‐terminal location, whereas the transactivator domain, followed by the DNA binding domain with zinc‐finger motifs, are N‐terminal [8]. The AR gene consists of eight exons, but several alternative transcripts with different influences on the cell have been described [8]. One of the most studied is the AR‐V7 transcript, which due to cryptic exon 3 splicing lacks the ligand‐binding domain and can transduce the signal independently from androgens [8, 9, 10].

A recent pan‐cancer study revealed that AR is overexpressed in GBM [11], and few studies explored AR as a possible treatment target [10, 12]. The use of antiandrogens *in vitro*, e.g., enzalutamide, led to inhibition of proliferation of GBM cells [10, 12, 13]. In mouse models, the antiandrogen treatment reduced the cancer stem cell population and tumour growth, which was further enhanced by radiation [12, 13]. Additionally, treatment of GBM cell lines with DHT seems to inhibit transforming growth factor β (TGFβ) signalling, which has been shown to partially act as tumour suppressor in GBM [14]. Hence, the use of antiandrogens could potentially restore the tumour suppressive activity of TGFβ in patients with high levels of DHT, thereby contributing to a positive therapeutic effect.

There are a limited number of studies exploring AR antagonists as possible treatment for GBM, and not much is known about AR genetic and epigenetic characteristics in GBM, which could influence the receptor's activity. Mutation frequency in AR in GBM is very low, only about 1%, as found in cBioPortal v3.7.5 based on The Cancer Genome Atlas (TCGA) data [15, 16]. Hence, mutation analysis is not the aim of this study. However, copy number (CN) alterations of AR seem to be a common event in GBM, influencing gene expression [10]. Additionally, there is a polymorphism in the AR gene, which directly relates to the function of the receptor [17]. A stretch of polyglutamine‐coding CAG repeats in exon 1 of the AR gene ranges from 6 to 39 repeats in healthy individuals, and the length inversely correlates with the transactivation capacity of AR [18]. Moreover, it has been reported that lower numbers of CAG repeats increase the risk of prostate cancer [19]. Finally, hypomethylation of the AR promoter region can lead to increased transcription of the gene. There are several CpG sites that seem to be of importance for AR expression regulation, as shown, for example, in the mutation‐negative androgen insensitivity syndrome [20]. We sought to analyse these characteristics to gain a better understanding of AR function in GBM and to investigate whether AR expression or CN alterations influence overall survival (OS).

## 2 Materials and methods

### 2.1 Study subjects

**Fig. 1:**
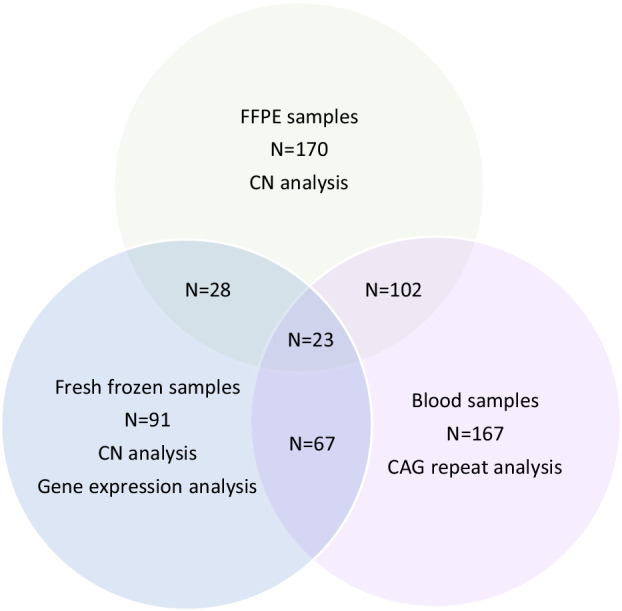
Graphical representation of samples from GBM patients collected at Linköping University Hospital and the overlap of cohorts. Each circle represents one cohort and contains information about the analyses performed.

All patients included in this study underwent surgery at Linköping University Hospital, were at least 18 years old, and provided written informed consent. The study was conducted according to the guidelines of the Declaration of Helsinki and approved by the Regional Ethics Committee of Linköping University, Sweden (M167‐07, 2010/76‐32, 2012/131‐32, 2012/368‐32, 2015‐362‐32). All patient samples, as well as control samples were collected in South‐East Sweden. Table 1 and Fig. 1 contain additional cohort information. The first cohort consisting of 179 GBM isocitrate dehydrogenase (IDH) wild‐type formalin‐fixed paraffin‐embedded (FFPE) samples was described previously [21]. All patients received postoperative temozolomide concomitant with radiotherapy and DNA, available for 170 samples, was used for CN analysis of AR.

**Table 1:** Patient cohorts used in the study.

Cohort	Total	Females (%)	Males (%)	Mean age ± SD (years)
FFPE	170	64 (37.6)	106 (62.4)	58 ± 8
Fresh‐frozen GBM	91	32 (35.2)	59 (64.2)	62 ± 12
Blood samples	167	61 (36.5)	106 (63.5)	60 ± 10
TCGA GBM	354	135 (38.1)	219 (61.9)	61 ± 13
IDH‐mutated fresh‐frozen samples	83	37 (44.6)	46 (55.4)	47 ± 14
TCGA IDH‐mutated	226	96 (42.5)	130 (57.5)	41 ± 12

The second cohort consisted of 91 fresh‐frozen samples, partially overlapping with the FFPE cohort (Fig. 1). These samples were used for CN, gene expression and AR promoter methylation analysis. DNA and RNA were extracted with AllPrep DNA/RNA Mini Kit (Qiagen, Hilden, Germany), and all samples were sequenced to confirm IDH wild‐type status, as described in [21]. Treatment and survival data was available for part of this cohort.

For comparison, in a third cohort of 83 IDH‐mutated fresh‐frozen gliomas processed as mentioned above, CN and gene expression analysis were also performed.

For the analysis of CAG repeats in exon 1 of AR, we used matched blood samples from the previous two cohorts and 21 additional samples for which tumour sample was not available (Fig. 1). Blood samples from healthy individuals from the same region of Sweden as the GBM patients were used as controls. These were from a random sample collection that was carried out between 1998 and 2 000 and included 401 females and 398 males with a mean age of 46 ± 17 years.

As an additional cohort, we included primary IDH‐wild type GBM from TCGA (Table 1). Clinical and transcriptome microarray data for these patients were accessed via the GlioVis portal [22, 23]. Reverse phase protein array protein expression data (level 4) of 150 patients from this cohort was also accessed via The Cancer Proteome Atlas (TCPA) [24, 25]. For comparison, we included a group of 226 IDH‐mutated tumours with RNA‐seq data from TCGA, also accessed via the GlioVis portal.

### 2.2 Genotyping of AR

For all samples included in the CAG repeat analysis, DNA was extracted from blood using Maxwell 16 Blood DNA Purification Kit (Promega, Madison, WI, USA) on the Maxwell 16 Instrument (Promega). A fragment encompassing the variable region was amplified with primers: forward‐ACCGAGGAGCTTTCCAGAAT, reverse‐ GCTGCCTGGGGCTAGTCTCTT, and sequenced for 20 healthy males from the control cohort. Amplification of exon 1 of AR was done with MyTaq DNA Polymerase (Bioline Reagents, London, UK), and a touchdown protocol was applied. The first three cycles were performed with annealing temperature 63 °C, followed by three cycles with annealing temperature 62 °C and 29 cycles with annealing temperature 61 °C. Sequencing was performed according to the BigDye Terminator v3.1 (Applied Biosystems, Vilnius, Lithuania) protocol with fragment separation on 3500 Genetic Analyzer (Applied Biosystems, Hitachinaka, Japan). Electropheograms were visualised in the sequence scanner Software 2 (Applied Biosystems) and aligned using blast to confirm correct amplification and enable counting of CAG repeats. Eight samples with a different number of repeats were selected and used as controls and markers in the following fragment analysis. DNA extracted from blood of 799 healthy controls and 167 GBM patients was amplified using the same set of primers as for sequencing but with the forward primer labelled with FAM fluorochrome at the 5′ end. Samples were then diluted 1 : 12 with water, and 1 μL was mixed with 0.3 μL of GeneScan 600 LIZ dye Size Standard (Applied Biosystems) and 15 μL of Hi‐Di Formamide (Applied Biosystems, Warrington, UK), and separated in the 3500 Genetic Analyzer. Fragments were analysed with the genemapper v.4.1 (Applied Biosystems) and the number of CAG repeats was determined by comparison of fragment sizes to control samples included in every run.

### 2.3 Methylation analysis

Methylation analysis of 17 CpG sites spanning 750 bp of the promoter region of AR in 91 fresh‐frozen tumour samples (32 females and 59 males) was done by pyrosequencing. EZ DNA Methylation Kit (Zymo Research, Irvine, CA, USA) was used for bisulfite conversion of 350 ng of DNA, out of which 20 ng was used for each of the subsequent amplification reactions done with the HotStarTaq Master Mix Kit (Qiagen). The promoter region was divided into seven amplicons and all used primers adapted from Hornig et al. [20], or designed in psq assay design 2.0.1.15 (Qiagen), are given in the Table S1, together with the amplification conditions. Successful PCR was confirmed with the QIAxcel (Qiagen) capillary electrophoresis, followed by pyrosequencing on the PyroMark MD (Qiagen) using the PyroMark Gold Q96 CDT (Qiagen) reagents as per manufacturers' protocol. Dispensation orders for each amplicon can be found in the Table S1. Obtained results were analysed using pyroq‐cpg 1.0.9 software (Biotage, Uppsala, Sweden).

### 2.4 Copy number and gene expression of AR

The AR CN analysis was performed on 91 fresh‐frozen GBM and 83 IDH‐mutated gliomas, as well as 170 FFPE GBM samples using droplet digital PCR. The same technique was used for assessment of mRNA expression, both for total AR as well as the AR‐V7 variant. In all instances, droplet generation was done using the Automated Droplet Generator (BioRad, Hercules, CA, USA), and after PCR, droplet reading was performed on the QX200 Droplet Reader (BioRad). The results were analysed using the quantasoft v1.7.4 (BioRad).

For CN analysis, DNA from fresh‐frozen tissue was subjected to enzymatic digestion (HaeIII; ThermoFisher Scientific, Vilnius, Lithuania). This step was omitted for DNA from FFPE tissue due to its pre‐existing fragmentation. Then, 20 ng of DNA was mixed with probes and ddPCR Supermix for Probes (no dUTP) (BioRad), and amplified according to the manufacturer's protocol with annealing temperature of 60 °C. The FAM‐labelled probe targeting AR (dHsaCP2500359; BioRad) was used together with the reference HEX‐labelled probe AP3B1 (dHsaCP2500348; BioRad). Deletion was reported for CN values below or equal to 1.7 for females and 0.7 for males, amplification for females was reported for CN values above 2.3 and 1.3 for males.

Reverse transcription of 500 ng–1 mg RNA for expression analysis was done with Maxima First Strand cDNA Synthesis Kit for RT‐qPCR (ThermoFischer Scientific). We used primers and probes for AR gene expression, as reported by Ma et al. [9] and GUSB as a reference gene (FAM‐labelled probe, Hs99999908_m1; ThermoFisher Scientific). The ddPCR Supermix for Probes (no dUTP) was mixed with cDNA, primers and probes, and amplified in the PCR with annealing temperature of 60 °C. For total AR, cDNA equivalent of 5 ng of RNA was used and 50 ng for the AR‐V7 analysis. Total AR was normalised with GUSB expression, and AR‐V7 expression was compared with the total AR expression before further statistical analysis.

### 2.5 Gene set enrichment analysis

The gene expression data from TCGA GBM samples were used in the gene set enrichment analysis (GSEA), keeping the analysis separate for males and females. The analyses included hallmark gene sets version 7.4 and was performed on gsea 4.1.0 software [26, 27]. In the first analysis with the gsea software default settings, high and low AR expressions were treated as categorical values, and phenotype labels for the two groups were created based on the survival analysis results. In the second analysis, the AR expression values were treated as continuous data, allowing us to look for enrichment of gene sets associated with the increasing AR expression within each sex group, without the influence of survival data. Here, the Euclidean distance as a metric for ranking genes was used. We also investigated the enrichment of microRNA (miR) targets gene sets associated with AR expression due to the involvement of AR in miR regulation. In all analyses, only gene sets with false discovery rate (FDR) < 25% and *P* < 0.01 were considered significant.

### 2.6 Statistical analysis

Differences in OS were analysed with the Kaplan–Meier log rank method complemented by maximally selected rank statistics [28] used for cut‐off selection for gene expression data from TCGA. Results were considered significant when *P* < 0.05. All statistical analyses were done in spss v.26 (IBM, Armonk, NY, USA) unless stated otherwise, and maximally selected rank statistics was done in rstudio v.1.4 (Boston, MA, USA) with r version 4.0.3 using packages maxstat and survminer. Normality of distribution of CAG repeats, AR gene expression and methylation data were tested with the Shapiro–Wilk test before other tests were performed. The Kruskal–Wallis test with Bonferroni correction for multiple pairwise comparisons was used to compare AR gene expression from TCGA data and between fresh‐frozen GBM and IDH‐mutated gliomas, whilst the Mann–Whitney *U* test was used for 2‐group AR mRNA expression between females and males. The Pearson coefficient was used for correlation analysis between AR CN and gene expression. The CAG repeats were compared with Mann–Whitney *U* test. In females, two alleles were separated (into shorter and longer alleles) before statistical analysis, but we also compared the biallelic mean values. Additionally, we dichotomised data based on the median value from the control group (biallelic median value was used for females) and divided into a group with lower and equal or higher number of CAG repeats [29]. Such groups were compared with the χ^2^ test.

## 3 Results

### 3.1 Copy number changes and gene expression of AR

First, we analysed CN changes in FFPE and fresh‐frozen GBM samples, and detected amplifications and deletions of the gene. In both cohorts, combined CN alterations were more frequent in females than in males (Table 2). More frequent CN alterations, though only deletions, were also observed in females in IDH‐mutated gliomas.

**Table 2:** Frequencies of *AR* CN changes in the GBM cohorts.

Cohort	Sex	Amplification (%)	Normal CN (%)	Deletion (%)	Missing
FFPE samples	Females	12 (18.8)	37 (57.8)	15 (23.4)	0
	Males	13 (12.3)	88 (83)	5 (4.7)	0
Fresh‐frozen samples	Females	4 (12.4)	25 (78.1)	3 (9.4)	0
	Males	5 (8.5)	53 (89.8)	0	1 (1.7%)
IDH‐mutated glioma fresh‐frozen samples	Females	0	32 (86.5)	5 (13.5)	0
	Males	1 (2.2)	45 (97.8)	0	0

**Fig. 2:**
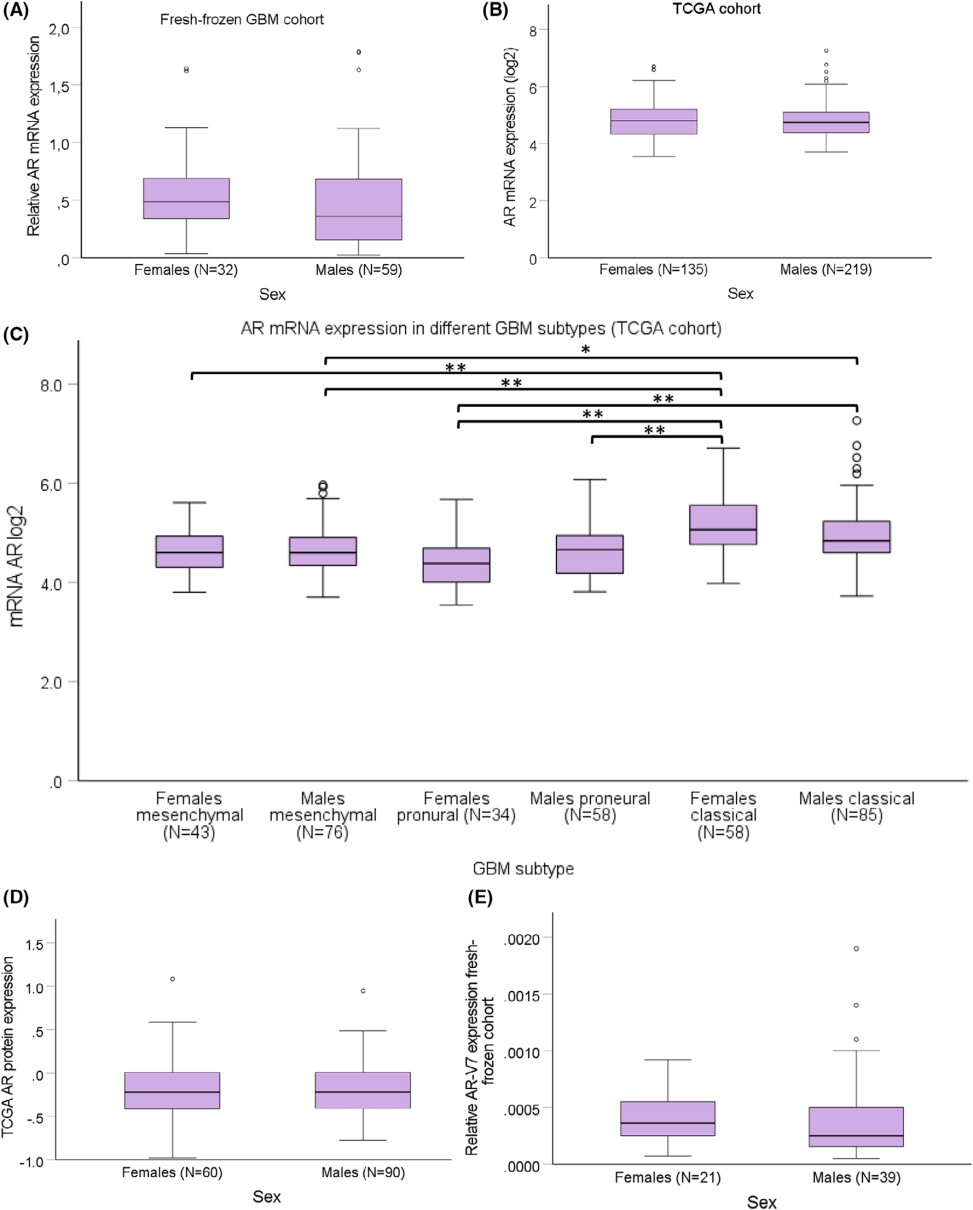
Comparison of *AR* expression in females and males with GBM from different cohorts. There were no differences in the expression of *AR* between the sexes in the Linköping fresh‐frozen cohort (*N* = 91) (A) and TCGA cohort (*N* = 354) (B) evaluated with Mann–Whitney *U* test. The highest *AR* mRNA expression was found in the classical subtype of TCGA cohort analysed with Kruskal–Wallis test and Bonferroni correction for multiple pairwise comparisons but no differences between sexes were found (C). Mesenchymal and proneural subtypes did not differ from each other and no sex differences were observed. There were neither any differences in the AR protein expression in samples from females and males in TCGA cohort (D), nor in the AR‐V7 transcript expression in the Linköping fresh‐frozen cohort (E), compared with Mann–Whitney *U* test. Error bars represent 95% confidence interval; *P < 0.05; **P < 0.005.

We then looked at the AR gene expression in the fresh‐frozen cohort and no differences were observed between the expression in males and females (*P* = 0.099) (Fig. 2A). Correlation analysis between AR CN and mRNA expression in fresh‐frozen samples revealed that a positive correlation was present in male GBM samples (Pearson coefficient 0.3, *P* = 0.022). In female GBM, no such relationship was found (Pearson coefficient −0.130, *P* = 0.479), however, in IDH‐mutated gliomas the correlation was the opposite, found in females but not in males (Table 3). We did not detect AR gene expression differences between sexes in TCGA (Fig. 2B). However, GBM subtypes are characterised by different molecular alterations, hence, TCGA samples were divided by subtype into proneural, mesenchymal and classical tumours, introduced by Wang et al. [6], and AR gene expression was compared. We found that significantly higher expression of AR is present in the classical subtype in comparison with proneural (*P* = 2 × 10^−8^) and mesenchymal (*P* = 1 × 10^−6^) subtypes. Additionally, we divided samples by sex, and multiple pairwise comparison revealed significant differences between several subtypes and across sexes, but no differences were found between females and males within the same subtypes (Fig. 2C). Protein AR expression also did not differ between sexes in TCGA samples (*P* = 0.965) (Fig. 2D). Interestingly, there were also no differences between AR mRNA expression in males and females with IDH‐mutated gliomas and GBM fresh‐frozen samples (Fig. S1).

**Table 3:** The Pearson correlation analysis between CN and gene expression of *AR*.

Sample type	Pearson correlation	Number of samples (*N*)
GBM, females	−0.130; *P* = 0.479	32
GBM, males	**0.300; *P* = 0.022** *	58
IDH‐mutated, females	**0.363; *P* = 0.029** *	36
IDH‐mutated, males	0.202; *P* = 0.212	40

The significant data are indicated in bold.

* *P* < 0.05.

Next, we undertook the analysis of the AR‐V7 transcript variant, which was limited to 64 fresh‐frozen samples due to high RNA demand for reliable transcript detection. This already indicated low expression of AR‐V7 transcripts in the tumours measured in relation to total AR. All of the 64 analysed samples were positive for AR‐V7 and its fraction of total AR expression did not differ between males and females (*P* = 0.258) (Fig. 2E).

### 3.2 The association of AR expression and overall survival

We further investigated the influence of AR CN and gene expression on OS. First, we analysed the cohort of FFPE samples, with the advantage of all patients following an equal treatment regimen, and we compared survival between patients with AR amplification, deletion and normal CN with the log rank Kaplan–Meier analysis. Males and females were analysed in combination and separately, but no significant differences were found. There were also no survival differences in the analyses of amplification vs. remaining samples, in the sex‐combined and sex‐separated setting (Fig. S2).

**Fig. 3:**
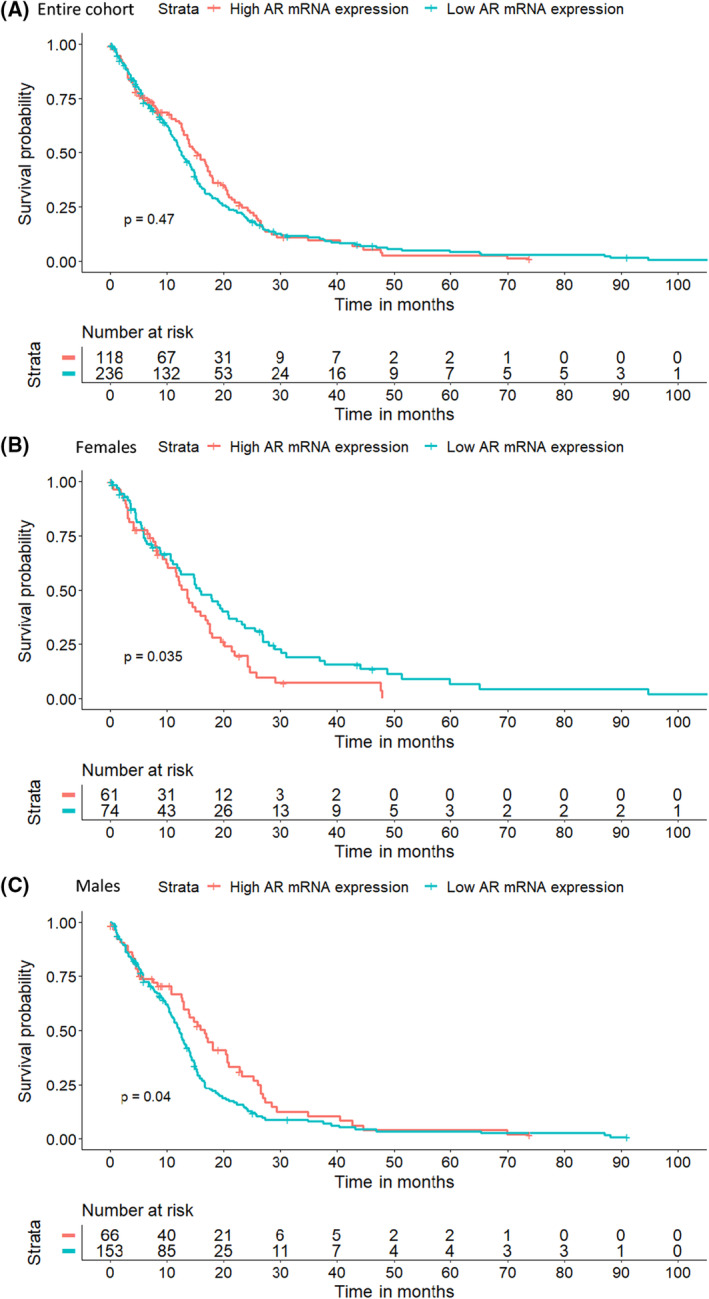
Survival analysis of TCGA patients with tumours with high and low *AR* gene expression. Kaplan–Meier log‐rank survival analysis was performed on TCGA data for the entire cohort (*N* = 354) (A), for females (*N* = 135) (B), and for males (*N* = 219) (C), and showed the opposite influence of *AR* mRNA expression on OS depending on sex.

Due to a lack of correlation between CN and gene expression in fresh‐frozen female GBM, we decided to extend the analysis and check for an association between mRNA expression and survival. Interestingly, in the univariate analysis conducted in TCGA we found a significant influence of AR expression on OS but only after the separation of sexes (Fig. 3). Before Kaplan–Meier log rank analysis, cases were assigned to two groups based on the expression (log_2_) cut‐off value estimated with maximally selected rank statistics ensuring the best separation of survival curves and allocation of at least 20% of samples in each group. In females, the cut‐off value was 4.8303log_2_, and in males it was estimated at 4.9594log_2_. Surprisingly, we recorded the opposite effect of AR expression on survival with high AR mRNA expression associating with better survival among males (12.2 vs. 16.6 months, *P* = 0.04) (Fig. 3C), and worse survival among females (13.6 vs. 15.7 months, *P* = 0.035) (Fig. 3B). No significant association between AR gene expression and survival for males or females was found in IDH‐mutated tumours from TCGA (Fig. S3).

### 3.3 AR promoter methylation in GBM

**Fig. 4:**
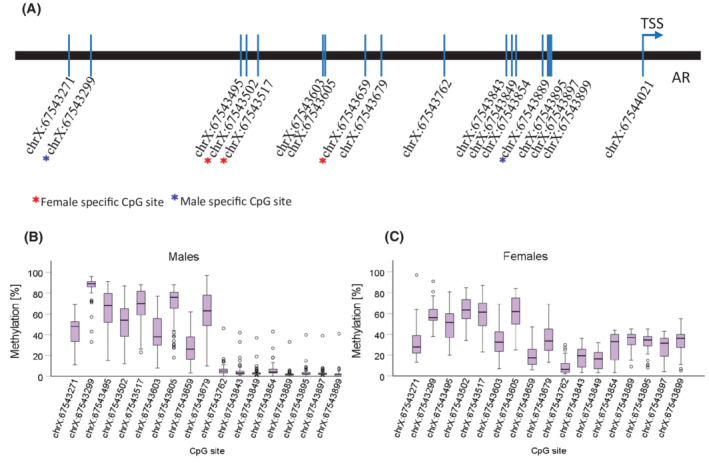
Results of the methylation analysis of the *AR* promoter region of 91 GBM samples from the fresh‐frozen Linköping cohort. Seventeen CpG sites located upstream from the TSS were analysed with pyrosequencing (A). Horizontal bars mark the median methylation value at each CpG site in males (*N* = 59) (B), and females (*N* = 32) (C). Boxes depict first and third quartiles, and whiskers are error bars with 95% confidence interval.

Pyrosequencing allows for semi‐quantitative evaluation of methylation at specific CpG sites. We analysed the promoter region of AR, with the first CpG site located 750 bases upstream from the transcription start site (Fig. 4A) in the cohort of fresh‐frozen samples. One of the X chromosomes in females is inactivated by methylation and such inactivation is believed to show no preference for the maternal or paternal allele. Hence, we expected higher methylation values in females throughout the entire analysed region. Surprisingly, we observed a tendency towards a lower average methylation in females (Fig. 4C) in comparison with males (Fig. 4B) in the region of chrX:67543271‐chrX:67543679, encompassing the first nine CpG sites (Table S2). At the remaining CpG sites, methylation was maintained at higher levels in females as predicted. Spearman correlation analysis of the methylation sites and gene expression of total AR revealed that methylation of different CpG sites correlates with AR gene expression in females and males (Table S3). In females, we found negative correlation between methylation of CpG sites at chrX:67543502, chrX:67543517 and chrX:67543659, and AR mRNA expression. Instead, the negative correlation in males was found for chrX:67543299 and chrX:67543895 sites, suggesting sex‐dependent regulation of AR expression.

### 3.4 AR‐based gene set enrichment analysis

**Fig. 5:**
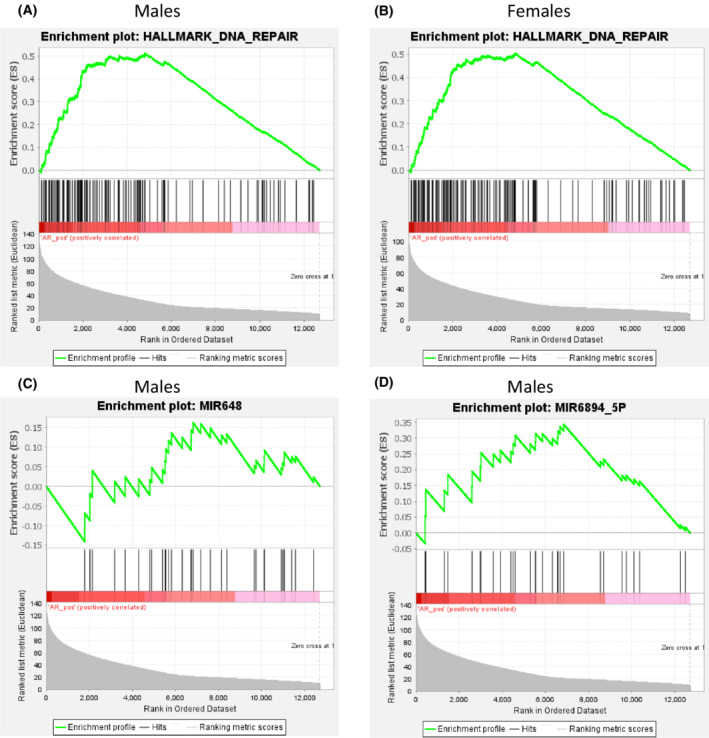
Results of the gene set enrichment analysis performed on TCGA data. The DNA repair gene set was significantly enriched in *AR* expressing male samples (*N* = 219) (A), but not in females (*N* = 135) (B), where the FDR < 25% and *P*‐value < 0.05 were not reached. Enrichment among miR targets associated with *AR* expression was found only in males and it concerned miR648 (C) and miR6894‐5p (D). These analyses were performed with the gsea tool and default settings.

We performed GSEA for TCGA cohort, trying to resolve cellular mechanisms that are responsible for the sex difference linking AR expression with survival, however, no gene sets were identified. We then repeated GSEA, but instead of dividing samples into two groups based on their survival, we looked for gene sets associated with AR expression (AR‐positive samples). Surprisingly, only one set of genes, namely DNA repair genes, was associated with AR expression in males (Fig. 5A). In females, the same gene set appeared among the top scored sets, but the FDR value did not meet the threshold value (Fig. 5B), indicating that these genes may be of less importance for females. Gene sets enriched among miR targets associated with AR expression were identified only in males and statistically significant were targets for miR648 and 6894‐5p (Fig. 5C,D).

### 3.5 CAG genotyping

**Fig. 6:**
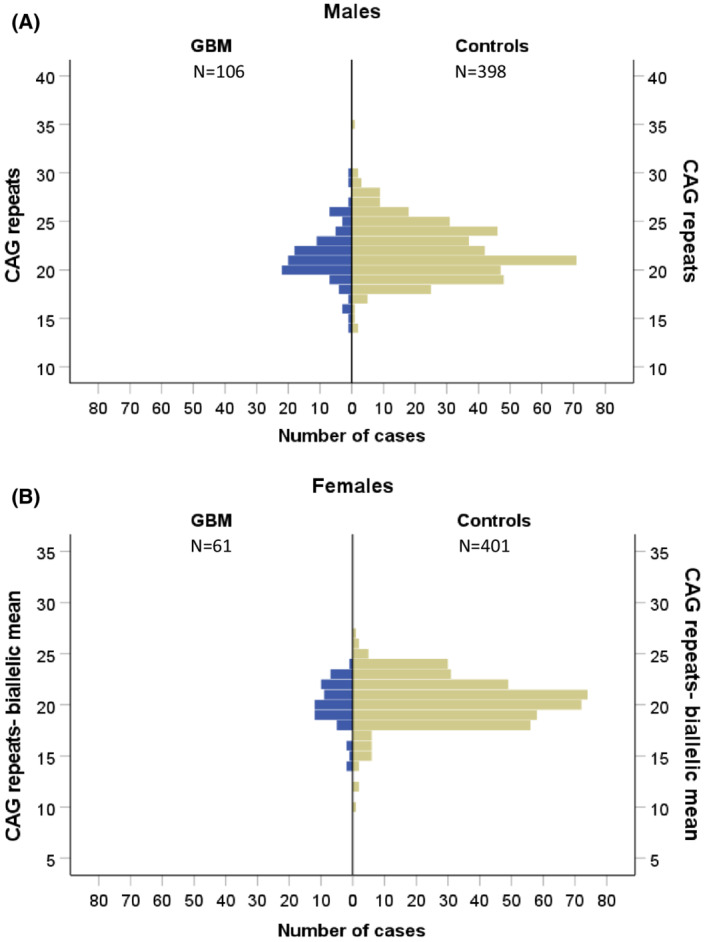
Comparison of the number of CAG repeats in exon 1 of *AR* in GBM and healthy controls. Results from one allele for males (A), and the CAG biallelic mean values for females (B) showed no distribution differences between the patients (blue) and healthy population (green).

The mean length of the polyglutamine chain in exon 1 of AR in male GBM patients was 21.4 (±2.7), and among control males 21.9 (±2.8), whereas for the female patients it was 20.1 (±2.2) for the shorter allele and 23.4 (±2.6) for the longer allele, and in female controls 20.4 (±2.3) and 23.6 (±2.4), respectively. The number of repeats ranged from 14 to 35 in males and from 10 to 31 in females. Among the samples tested, we found 62 (15.5%) homozygous females in the control group and 15 (24.6%) in the GBM group but the difference between groups was not significant (χ^2^, *P* = 0.075). We did not observe any significant differences between the CAG length distributions in either sex, even after treating data as binary qualitative and dividing samples based on the median CAG repeat value in the control group (Fig. 6), this pointing to a lack of relationship between number of CAG repeats and the GBM diagnosis.

## 4 Discussion

The results presented in this study complement the current knowledge about AR and its involvement in GBM, also indicating a sex‐specific influence of AR on patients' survival. The abundance of AR expression in GBM is encouraging as it may constitute a novel therapeutic target. In fact, enzalutamide, an oral AR antagonist used for prostate cancer treatment [30], was shown to induce apoptosis in several GBM cell lines and decrease tumour growth in mouse models [10, 12, 13, 31]. The association between AR expression and patients' survival was previously studied in TCGA datasets, where high AR mRNA and protein expression was a negative prognostic factor in low‐grade glioma [11], a finding we could not confirm when analysing IDH mutated glioma. In the same study, a positive influence of high AR expression on survival in cancers such as acute myeloid leukaemia or cutaneous melanoma was documented, highlighting a dual effect that AR can exhibit depending on the nature of the malignancy. However, there was no association between total AR expression and survival reported for GBM patients. The results only focusing on the entire cohort and possibly also different dichotomisation approaches could have contributed to the differences in the reported outcomes. Here, we analysed females and males both in combination and separately in contrast with the analysis done by Hu et al. [11]. We also used maximally selected rank statistics instead of median expression value, which ensures finding a data‐driven cut‐off point. By using such approach, we found a sex‐specific association between AR gene expression and survival in TCGA data, but we could not determine the role of CN alterations for this cohort. At the same time, we found a positive correlation between gene expression and CN in male fresh‐frozen GBM samples, which prompted us to the analysis of association between CN and survival in FFPE cohort, yet no association was found. This might be a result of intrinsic differences between cohorts, such as clinical factors and treatment. The role of these will need additional analysis in the future. Patients with GBM often undergo treatment with an alkylating agent, commonly temozolomide, and/or radiation therapy [32]. A large part of cancer treatments is based on causing an accumulation of DNA damage that is beyond the cell's capacity to repair, which forces the cells into apoptosis. Here, we report on an association between AR expression in GBM and enrichment in the expression of DNA damage response genes, especially enhanced in males. This finding is reinforced by the positive correlation of protein expression of AR and several proteins involved in DNA damage response in both sexes (Table S4), which aligns with the results reported by Werner et al. [12]. There, AR inhibitors radiosensitised GBM cells in *in vitro* and *in vivo* models, by downregulating DNA damage response transcriptional programs. The tumour suppressor 53 (p53) acts as a transcription factor and the main controller of cell cycle in response to DNA damage [33]. It has been shown that p53 can negatively regulate AR expression, e.g., in prostate cancer [34]. Additionally, recent findings showed sex‐specificity of p53 mutations in GBM, as well as differences in the effects induced by the same mutation in cells of different sex [35]. As reported, this is likely due to the binding of mutated p53 at different genomic localisations depending on the type of mutation and sex of the cells. Interestingly, some of these binding sites were also recognised as AR‐binding sites, and p53 could either enhance or inhibit the AR‐mediated effects, introducing even greater differences between males and females. It has also been shown that in normal tissues’ transcription factors, despite the same expression, activate different transcriptional programs in males and females [36]. In other cancer forms, especially prostate cancer, AR has been shown to regulate numerous miRs [37, 38, 39, 40]. Although transcriptome data used here is lacking information about the levels of expression of miR themselves, we were able to identify enrichment on targets for miR648 and miR6894‐5p, which were positively associated with AR expression in males. Interestingly, miR648 was previously found to disturb translation of *O*‐6‐methylguanine‐DNA methyltransferase (MGMT) [41]. Silencing of MGMT through methylation predicts the response to the alkylating agent temozolomide, commonly used for GBM treatment, this leading to extended OS of patients [42, 43]. The decreased translation of MGMT mediated by AR‐dependent expression of miR648 could explain the correlation between high AR expression and longer survival of males with GBM.

Glioblastoma presents to be a tumour dependent on other sex hormone receptors as well. Intriguingly, oestrogen receptor beta was shown to act as a tumour suppressor in GBM by downregulating DNA damage response [44], which confers the opposite effect to AR signalling. Enhanced oestrogen receptor beta expression similarly to AR inhibition, sensitises GBM to treatment [44]. This indicates the possibility of estrogenic protection against GBM development in women until the postmenopausal decline of the hormone [45]. In a different study, progesterone was reported to inhibit the glycolytic metabolism in GBM, as well as the EGFR/PI3K/Akt/mTOR signalling [46], highly active in GBM. This could sufficiently contribute to the sex difference. Hence, the interplay between sex hormones and their receptors could be partially responsible for the sex differences observed in GBM, including the AR‐dependent survival differences presented here.

We also report on the frequency of AR CN changes, which affect females more often than males but without a clear direction towards amplifications or deletions. In the previously published work by Zalcman et al. [10], AR amplification was reported in 27% of men and 38.2% of women, whilst we found amplifications in 8.5% and 12.3% of males and 12.4% and 18.8% of females, depending on the GBM cohort. Zalcman et al., reported AR deletions only for females (28.5%) [10] but we found deletions in 4.7% of the male GBM samples and up to 23.4% in females. We also found that CN alterations were more frequent in female IDH‐mutated gliomas. A higher frequency of genetic aberrations on the X chromosome in females compared to males is common in tumours, including gliomas [47, 48, 49, 50]. Furthermore, expression of mutant alleles is limited in females, as they are often found on the inactivated X chromosome, providing additional protection against cancer development [48], and likely contributing to the overall sex imbalance observed in cancer [51]. At the same time, epigenetic inactivation of one of the X chromosomes may be locally disrupted, leading to increased expression of genes that have escaped from such inactivation. In cancer, this is especially important for tumour suppressors, e.g., ATRX, DDX3X [52]. Interestingly, one study reported on skewed X chromosome inactivation in blood of females 40 years old or younger with high‐grade glioma in comparison to healthy controls, pointing to non‐random inactivation of the X chromosome as a risk factor [53]. In our study, no sex differences were observed in the expression of AR in the tumours, prompting us to the conclusion that the AR gene does not escape X chromosome inactivation in GBM.

As reviewed by Bramble et al. [54], neural stem cells from the subventricular zone (SVZ) of mice and rat embryos, as well as adult animals, express AR. GBM is proposed to originate from the neural stem cells of the SVZ [55]. Additionally, a sex difference in the proliferative response to the treatment with concomitant testosterone and an AR inhibitor in adult murine neural stem cells has been reported, with XY cells proliferating despite the presence of an AR inhibitor [54]. These could be species‐specific effects, however, AR blockade in glioma cancer stem cells has been shown to decrease proliferation and downregulate markers related to stemness [13], making AR an even more interesting treatment target in GBM. Interestingly, a study based on a murine model of GBM revealed that male astrocytes with loss of neurofibromin 1 and p53 have higher tumourigenic potential than female astrocytes with the same aberrations [56]. The male astrocytes are also more likely to acquire a stem‐like cell phenotype [56]. A different study of mouse GBM models showed that sex differences in the potency to malignant transformation seem to be fuelled by the sex differences in cell cycle regulation and DNA repair [57]. Etoposide‐induced DNA damage was shown to trigger cell cycle arrest in female but not in male cells, which continued to proliferate despite higher number of chromosomal aberrations than in the female cells [57].

High AR expression in GBM has been previously reported by several groups [10, 11, 31, 58]. The AR‐V7 transcript variant is of particular interest in prostate cancer, where high expression correlates with worse prognosis and increasing resistance to antiandrogen treatment [8]. Zalcman et al. found expression of AR‐V7 in 30% of GBM [10], and we were able to detect this transcript in all analysed RNA samples, however, it represented only about 1% of the total AR expression. The limited AR‐V7 expression was also reported in the RNA‐seq data from TCGA [11]. This could indicate a need for AR ligands to activate downstream signalling in GBM. Interestingly, the ability to synthesise neurosteroids, including testosterone, in GBM cells, was recently reported by Pinacho‐Garcia et al. [59]. Under such conditions, the development of GBM would not be dependent on systemic androgens, though increased serum testosterone in glioma patients of both sexes have been reported [31].

In the methylation analysis, we found a sex difference in the CpG sites, where methylation inversely correlated with AR expression. Two CpG sites were identified for males and three different for females. Hornig et al. [20], showed that methylation at chrX67543495‐67543517 negatively correlated with AR expression in mutation‐negative androgen insensitivity syndrome in males, but here, we found two CpG sites located in this region, specific for females. This aligns with a previous report on methylation differences at single CpG sites in males and females with GBM by Johansen et al. [60]. However, we observed a drop in the methylation levels in the region from −259 to the TSS in both sexes, which could suggest that methylation over the promoter region is more important for AR expression than methylation at a single CpG. Apart from this, AR expression is also regulated by transcription factors binding to consensus sites [20], what seems to be in line with the lack of expression differences between males and females. It would require further studies to elucidate which transcription factors are essential for the AR expression in GBM.

To our knowledge, this is the first study exploring CAG‐length polymorphism in AR in GBM patients. A decreasing number of CAG repeats encoding polyglutamine was shown to be associated with an increased risk of prostate cancer [19], and longer alleles were associated with colorectal cancer and shorter survival of patients [61]. Moreover, in women with polycystic ovary syndrome, where androgenic signalling is strongly enhanced, shorter alleles are more common [29]. We hypothesised that in GBM cases the number of CAG repeats also would deviate from the normal population. However, we found no difference between patients and healthy individuals, and GBM risk does not seem to be associated with this short tandem repeat sequence.

## 5 Conclusions

In summary, sex differences are observed in the association of AR expression and survival as well as in the frequencies of AR CN alterations, but no association was found between CAG repeat number and GBM development. AR is commonly expressed in GBM of both sexes and the negligeable presence of the AR‐V7 transcript suggests the need for the ligand to trigger downstream signalling, which is more strongly linked to DNA damage response in males than in females. Hence, further research focusing on both mechanistic studies and discriminating sex differences associated with AR and other sex hormone receptors might translate into sex‐dependent treatment of GBM in the future.

## Conflict of interest

The authors declare no conflict of interest.

## Author contributions

MŁ and AM conceptualised the study, administered the project and performed data curation. MŁ designed the methodology, investigated and visualised the study, and wrote and prepared the original draft. MŁ and MT performed the formal analysis. MM, CB, MS, PM, MH and AM provided resources. MŁ, MT, MM, CB, MS, PM, MH and AM wrote, reviewed and edited the manuscript. AM supervised the study and acquired funding. All authors have read and agreed to the published version of the manuscript.

## Supporting information


**Fig. S1.** Relative expression of *AR* in the fresh‐frozen tumours collected in South‐East Sweden.
**Fig. S2.** Analysis of *AR* CN changes influence on overall survival in the FFPE Linköping cohort (Kaplan‐Meier log‐rank method).
**Fig. S3.** Survival analysis of IDH‐mutated tumours from TCGA.
**Table S1.** Primers used for the methylation analysis of the promoter region of *AR* and amplicon specific annealing temperatures.
**Table S2.** Mean methylation values for each analysed CpG site within the *AR* promoter region from the fresh‐frozen cohort.
**Table S3.** Results of Spearman correlation analysis between methylation of 17 CpGs in the promoter of *AR* and *AR* gene expression.
**Table S4.** Spearman's correlation of AR protein expression and 9 selected proteins; TCGA cohort (60 females and 90 males).

## Data Availability

The expression data that support the findings of this study are available in GlioVis, TCGA_GBM dataset at http://gliovis.bioinfo.cnio.es/ [22]. The protein expression data are available at The Cancer Proteome Atlas, TCGA, Glioblastoma multiforme dataset, https://tcpaportal.org/tcpa/ [24, 25]. The remaining data that support the findings of this study are available from the corresponding author (malgorzata.lysiak@liu.se) upon reasonable request.
