# Supplementary material for: The sex‐dependent role of the androgen receptor in glioblastoma: results of molecular analyses
Source: Mol Oncol. 2022 Jun 22;16(19):3436–51. doi: 10.1002/1878-0261.13262 (PMC9533693; doi:10.1002/1878-0261.13262)
Supplement: Supplementary file 1 — Fig. S1. Relative expression of AR in the fresh‐frozen tumours collected in South‐East Sweden. Fig. S2. Analysis of AR CN changes influence on overall survival in the FFPE Linköping cohort (Kaplan‐Meier log‐rank method). Fig. S3. Survival analysis of IDH‐mutated tumours from TCGA. Table S1. Primers used for the methylation analysis of the promoter region of AR and amplicon specific annealing temperatures. Table S2. Mean methylation values for each analysed CpG site within the AR promoter region from the fresh‐frozen cohort. Table S3. Results of Spearman correlation analysis between methylation of 17 CpGs in the promoter of AR and AR gene expression. Table S4. Spearman's correlation of AR protein expression and 9 selected proteins; TCGA cohort (60 females and 90 males). [file MOL2-16-3436-s001.docx]

**Supplementary Data**

**Supplementary Table S1.** Primers used for the methylation analysis of the promoter region of *AR* and amplicon specific annealing temperatures.

| Methylation site | Primers | Sequence to analyze | Dispensation order | Annealing temperatures |
| --- | --- | --- | --- | --- |
| chrX:67543271 | Amplicon 1:  F: TATTTTTGGGATAGTTTTAGTTGTAAAGT (5´biotin)  R: TCACCCAAACAAACAAATAAACACA  seq: AAAAACACTTACCATATACACAT | AAACRCTCAATTTTATCCAAAACTCTATAAACRTTTACTTTATATACAACCRCTTACTTTTCCAAATTACCTTCRCAT | CAGTCGACTCATTATCAACTCTATATCGATACTATATACATCGACTACTTCATACTACGACA | 58 ˚C, 3cycles; 57 ˚C, 3 cycles; 56 ˚C, 3 cycles; 55 ˚C, 31 cycles |
| chrX:67543299 |  |  |  |  |
| chrX:67543495 | Amplicon 2:  F: GTTGAGGGTTTTTAGAGTAAATGGT  R: CCATAACCAAACCAACAAATACTTAC (5´biotin)  seq: GGTTTTTAGAGTAAATGGTATA | ATGTTAYGAGGTTYGATTTATTTTTATGAYGGAA | TATGCTGATCGATGTCGATATTATGTATCG | 56 ˚C, 40 cycles |
| chrX:67543502 |  |  |  |  |
| chrX:67543518 |  |  |  |  |
| chrX:67543603 | Amplicon 3:  F: AGTAAGTATTTGTTGGTTTGGTTATG (5´biotin)  R: CCAAATTTAATTCCAAAACCCAATCTA  seq: TACCAAAAATTCTTTTCAAAACT | AATAAAAACRCRCAAATAAAAAAAT | CGATAATCGATCGACAT | 60 ˚C, 3cycles; 59 ˚C, 3 cycles; 58 ˚C, 3 cycles; 57 ˚C, 31 cycles |
| chrX:67543605 |  |  |  |  |
| chrX:67543659 | Amplicon 4:  F: AATTTTTGGTAGTTAGGAGTAGGT  R: CCAAATTTAATTCCAAAACCCAATCTA (5´biotin)  seq: TGGTAGTTAGGAGTAGGTA | TTTTTATYGTTTTTTTTTTTTTTTTTTYGTTTTTA | ATCTGATCTGTTCGTT | 56 ˚C, 40 cycles |
| chrX:67543679 |  |  |  |  |
| chrX:67543762 | Amplicon 5:  F: GGTTTTGGAATTAAATTTGGTGAGTG (5´biotin)  R: CCCCTACTTCCTAAATAACTCCTAC  seq: CTACTTTCCTAAACCAAAAT | TTAAACRCCAAAACT | ATGATCGACAAC | 56 ˚C, 40 cycles |
| chrX:67543843 | Amplicon 6:  F: GGGTTTTTTAGGGTTAGAGTTAG (5´biotin)  R: CTCCTTACCTTCCCACCTC  seq: TAACTTTAAAAAAACAAATACTAAC | RCAACRTAAACRAAAAC | CGACATCGATAGTCAAGAA | 60 ˚C, 3cycles; 59 ˚C, 3 cycles; 58 ˚C, 3 cycles; 57 ˚C, 31 cycles |
| chrX:67543849 |  |  |  |  |
| chrX:67543854 |  |  |  |  |
| chrX:67543889 | Amplicon 7:  F: GGAGTTTTGGAGTTTAAATTTTGGTTTAGG  R: CCTCCCCTTTCCTTTTCTC (5’biotin)  seq: TTGTTTTTTTAAAGTTATTAGGTA | GGYGTTAGYGYGYGGTGAGGGGAGGGGA | AGTCGTCATGTCAGTCTGTCGTG | 56 ˚C, 40 cycles |
| chrX:67543895 |  |  |  |  |
| chrX:67543897 |  |  |  |  |
| chrX:67543899 |  |  |  |  |


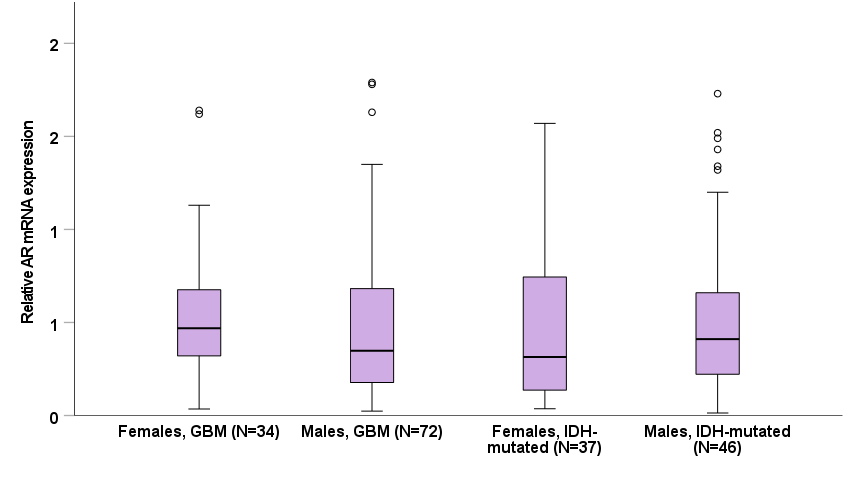


**Supplementary Figure S1.** Relative expression of *AR* in the fresh-frozen tumours collected in South-East Sweden. No significant differences in the expression between males and females of GBM or *IDH*-mutated gliomas were found (p<0.05).

A


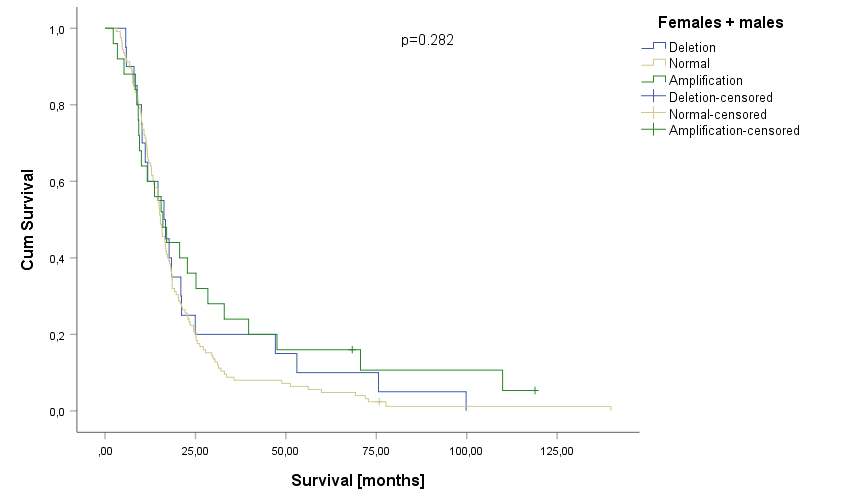


B
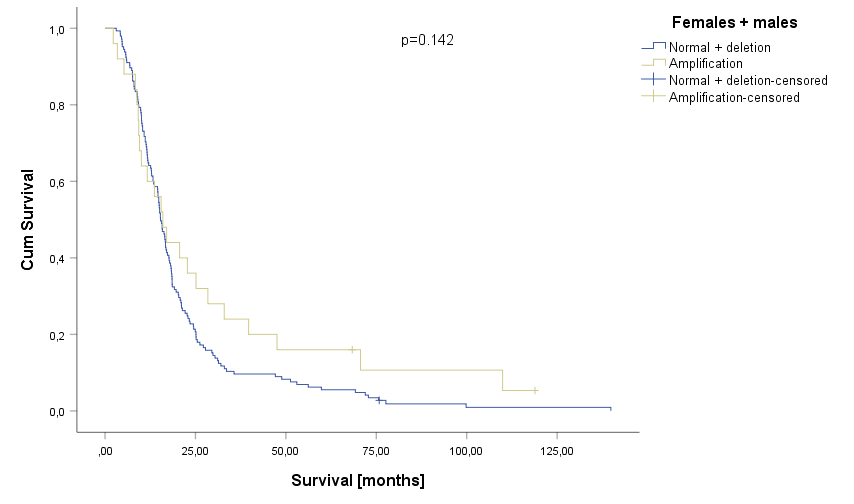


C


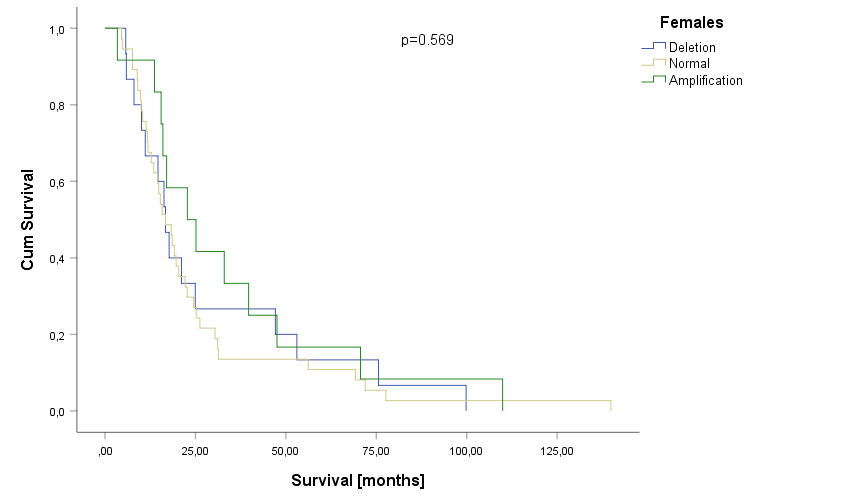


D
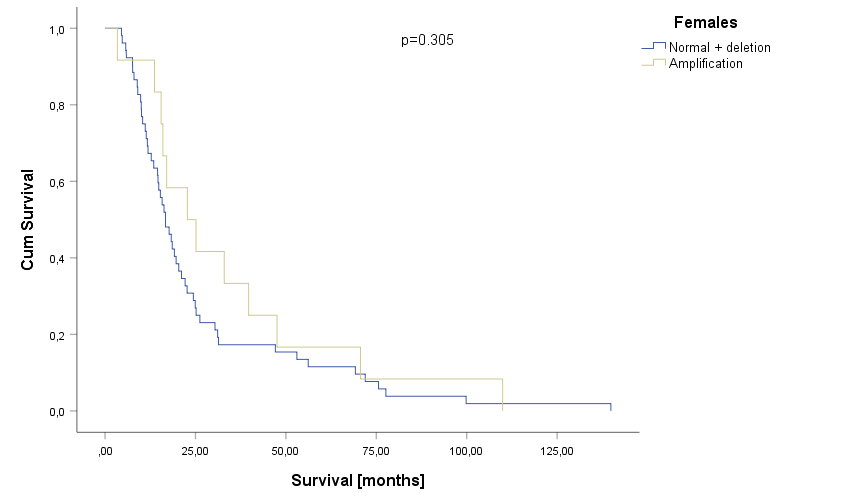


E
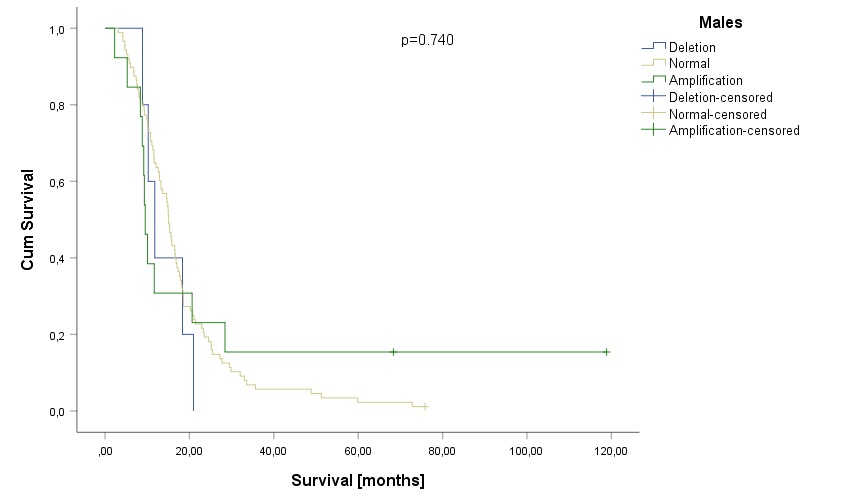


F
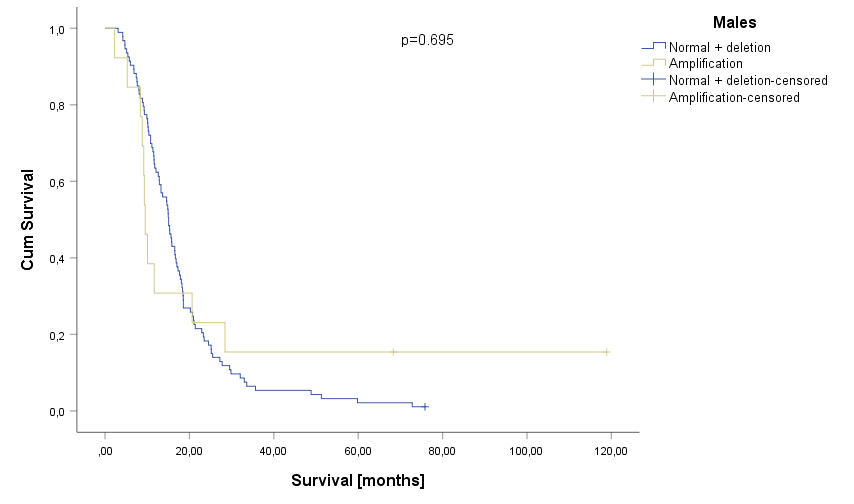


**Supplementary Figure S2.** Analysis of *AR* CN changes influence on overall survival in the FFPE Linköping cohort (Kaplan-Meier log-rank method). The curves in A-B are for the entire cohort (n=170), C-D for females (n=64) and E-F for males (n=106). Results from the figures A, C, E show the analyses of samples with normal *AR* CN vs. amplification of *AR* vs. deletion of *AR*. In figures B, D, F those with normal CN of *AR* and with deletions were combined and compared to samples with amplification.


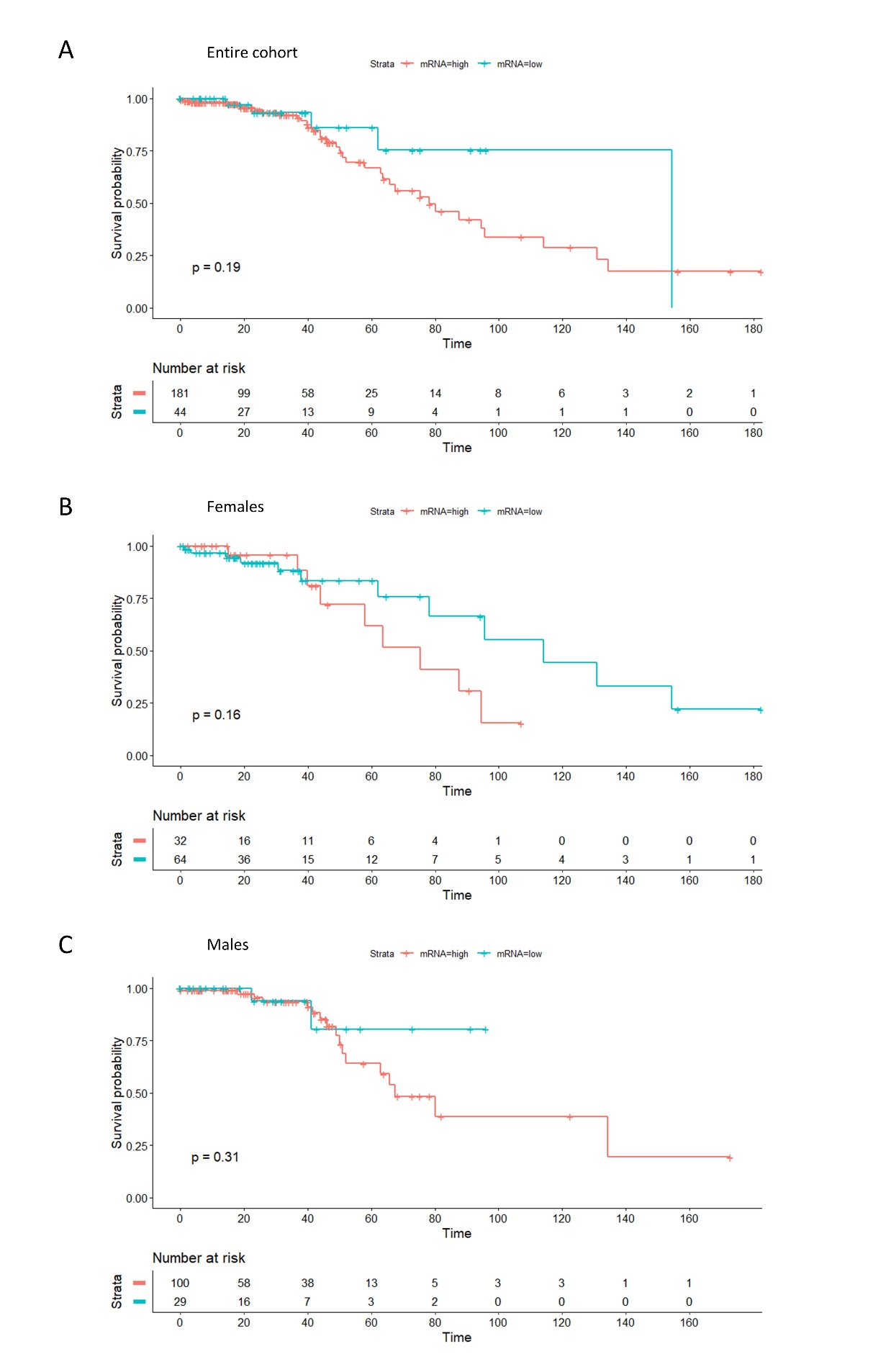


**Supplementary Figure S3.** Survival analysis of IDH-mutated tumours from TCGA. Samples were divided into low and high *AR* expression groups based on maximally selected rank statistics and Kaplan-Meier survival analysis was performed for the entire cohort (A), and separately for females (B) and males (C). None of the analysis showed significant difference between the low and high *AR*-expressing samples.

**Supplementary Table S2.** Mean methylation values for each analysed CpG site within the *AR* promoter region from the fresh-frozen cohort.

| **CpG site** | **Mean methylation value [%] ±SD** | |
| --- | --- | --- |
|  | **Females (n=32)** | **Males (n=70)** |
| AR1 chrX:67543271 | 32.6 ± 17.0 | 43.8 ± 13.0 |
| AR2 chrX:67543299 | 58.8 ± 10.7 | 85.8 ± 11.2 |
| AR3 chrX:67543495 | 48.8 ± 15.6 | 62.9 ± 21.5 |
| AR4 chrX:67543502 | 63.2 ±14.0 | 51.7 ± 18.6 |
| AR5 chrX:67543517 | 59.2 ± 16.0 | 67.2 ± 17.9 |
| AR6 chrX:67543603 | 35.3 ± 15.2 | 39.9 ± 18.5 |
| AR7 chrX:67543605 | 60.5 ± 16.2 | 69.0 ± 18.1 |
| AR8 chrX:67543659 | 19.6 ± 10.2 | 28.0 ± 14.4 |
| AR9 chrX:67543679 | 35.8 ± 14.6 | 60.8 ± 21.8 |
| AR10 chrX:67543762 | 9.2 ± 7.7 | 6.2 ± 6.5 |
| AR11 chrX:67543843 | 18.0 ± 9.2 | 5.2 ± 7.0 |
| AR12 chrX:67543849 | 15.7 ± 8.7 | 3.7 ± 5.2 |
| AR13 chrX:67543854 | 28.7 ± 13.5 | 6.1 ± 6.8 |
| AR14 chrX:67543889 | 33.9 ± 8.6 | 2.3 ± 4.3 |
| AR15 chrX:67543895 | 31.8 ± 9.9 | 3.6 ± 5.4 |
| AR16 chrX:67543897 | 27.8 ± 10.6 | 3.2 ± 5.0 |
| AR17 chrX:67543899 | 33.9 ± 11.3 | 2.5 ± 5.5 |

**Supplementary Table S3.** Results of Spearman correlation analysis between methylation of 17 CpGs in the promoter of *AR* and *AR* gene expression.

| **CpG site** | **Females (n=32)** | | **Males (n=59)** | |
| --- | --- | --- | --- | --- |
|  | **Correlation coefficient** | **p-value** | **Correlation coefficient** | **p-value** |
| AR1 chrX:67543271 | -0.129 | 0.480 | 0.048 | 0.717 |
| AR2 chrX:67543299 | -0.237 | 0.191 | 0.341 | **0.008*** |
| AR3 chrX:67543495 | -0.343 | 0.055 | 0.163 | 0.218 |
| AR4 chrX:67543502 | -0.427 | **0.015*** | -0.145 | 0.272 |
| AR5 chrX:67543517 | -0.510 | **0.003*** | 0.003 | 0.982 |
| AR6 chrX:67543603 | -0.070 | 0.703 | -0.034 | 0.797 |
| AR7 chrX:67543605 | -0.146 | 0.426 | 0.057 | 0.667 |
| AR8 chrX:67543659 | -0.367 | **0.039*** | -0.195 | 0.139 |
| AR9 chrX:67543679 | -0.182 | 0.319 | -0.012 | 0.929 |
| AR10 chrX:67543762 | -0.338 | 0.058 | -0.066 | 0.620 |
| AR11 chrX:67543843 | 0.069 | 0.708 | -0.134 | 0.313 |
| AR12 chrX:67543849 | 0.054 | 0.768 | -0.147 | 0.267 |
| AR13 chrX:67543854 | -0.049 | 0.789 | -0.109 | 0.412 |
| AR14 chrX:67543889 | 0.306 | 0.089 | -0.218 | 0.096 |
| AR15 chrX:67543895 | 0.163 | 0.374 | -0.275 | **0.035*** |
| AR16 chrX:67543897 | 0.227 | 0.211 | -0.156 | 0.238 |
| AR17 chrX:67543899 | 0.250 | 0.167 | -0.244 | 0.062 |

*p<0.05

**Supplementary Table S4.** Spearman's correlation of AR protein expression and 9 selected proteins; TCGA cohort (60 females and 90 males).

| **Gene/protein** | **All patients** | **Females** | **Males** |
| --- | --- | --- | --- |
| ATM | **0.253; p=0.002*** | **0.378; p=0.003*** | 0.133; p=0.210 |
| BRCA2 | **0.233; p=0.004*** | 0.184; p=0.159 | **0.244; p=0.02*** |
| CHK1 | **-0.266; p=0.001*** | -0.249; p=0.55 | -0.264; p=0.012 |
| CHK2 | -0.2; p=0.81 | -0.040; p=0.762 | -0.006; p=0.957 |
| EGFR | **0.291; p=0.000311*** | **0.385; p=0.002*** | **0.239; p=0.023*** |
| MSH2 | 0.123; p=0.133 | -0.1; p=0.941 | **0.21; p=0.047*** |
| MSH6 | 0.169; p=0.039 | 0.115; p=0.381 | 0.174; p=0.101 |
| P53 | 0.047; p=0.571 | -0.218; p=0.094 | **0.217; p=0.04*** |
| RAD50 | **0.414; p=0.0000001*** | **0.562; p=0.000003*** | **0.294; p=0.005*** |

*p<0.05
